# Supplementary material for: Comparison of Incidence and Prognosis of Myocardial Injury in Patients with COVID-19-Related Respiratory Failure and Other Pulmonary Infections: A Contemporary Cohort Study
Source: J Clin Med. 2023 Oct 8;12(19):6403. doi: 10.3390/jcm12196403 (PMC10573433; doi:10.3390/jcm12196403)
Supplement: Supplementary file 1 [file jcm-12-06403-s001.zip › jcm-2611523-supplementary.pdf]

Supplement Tables S1. Comparison of non-COVID-19 patients with versus without troponin checked

Features	Total	No troponin checked n = 18	Troponin checked n = 182	P value
Demographics				
Age (years)	60.5 (42;72)	54.5 (29.8;65.5)	62(44.8;73)	0.070
Gender				0.624
Male	92 (46)	7 (38.9)	85 (46.7)	
Female	108 (54)	11 (61.1)	97 (53.3)	
Body Mass Index (kg/m²)	25.9 (21.6; 31.3)	21.6(19.2;28.3)	26.6(22.3;31.3)	0.022
Comorbidities				
Hypertension	104 (52)	6 (33.3)	98 (53.8)	0.139
Diabetes mellitus	62 (31)	3 (16.7)	59 (32.4)	0.194
Chronic kidney disease	25 (12.5)	4 (22.2)	21 (11.5)	0.252
Cerebrovascular disease	26 (13)	1 (5.6)	25 (13.7)	0.478
Heart disease	51 (25.5)	2 (11.1)	49 (26.9)	0.168
Coronary artery disease	31 (15.5)	2 (11.1)	29 (15.9)	0.745
Heart failure	38 (19)	1 (5.6)	37 (20.3)	0.206
Valvulopathy	34 (17)	1 (5.6)	33 (18.1)	0.320
COPD	53 (26.5)	4 (22.2)	49 (26.9)	0.785
Smoking (present or past)	93 (46.5)	10 (55.6)	83(45.6)	0.465
Malignancy	26 (13)	5 (27.8)	21 (11.5)	0.065
HIV	17 (8.5)	2(11.1)	15 (8.2)	0.655
Laboratorial findings at ICU admission				
P/F ratio	206.7 (131;299)	234 (143;343)	203 (131;293)	0.350
White blood cell count (103/µL)	12 (8.8;16)	11.5 (7.5; 17.6)	12 (8.9;15.7)	0.752
Lactate (mmol/L)	1.7 (1.2; 3.4)	1.5 (1.1; 4.5)	1.8 (1.2; 3.2)	0.703
Creatinine (mg/dL)	1.2 (0.8;2)	0.8 (0.6; 1.8)	1.2 (0.8;2)	0.082
CRP (mg/L)	109 (36;212)	111 (52;211)	109 (35;213)	0.894
Outcome				
Death	54 (27)	6 (33.3)	48 (26.4)	0.580
Renal replacement therapy	22 (11)	1 (5.6)	21 (11.5)	0.699
Length of mechanical ventilation	6 (4;10.8)	6 (6;6)	6 (4; 11)	0.929

Data expressed as median (p25;p75) or n (%).

Supplement Tables S2. Comparison of COVID-19 patients with versus without troponin checked

Features	Total	No troponin checked n = 165	Troponin checked n = 1444	P value
Demographics				
Age (years)	59 (46;68)	60 (47.5;68)	58(46;68)	0.346
Gender				0.741
Male	891 (55.4)	89 (53.9)	802 (55.5)	
Female	718 (44.6)	76 (46.1)	642 (44.5)	
Body Mass Index (kg/m²)	30.4 (26.4; 35.6)	29.1(25.2;34.4)	30.4(26.5;35.7)	0.051
Comorbidities				
Hypertension	910 (56.6)	89 (53.9)	821 (56.9)	0.507
Diabetes mellitus	554 (34.4)	60 (36.4)	494 (34.2)	0.604
Chronic kidney disease	127 (7.9)	18 (10.9)	109 (7.5)	0.129
Cerebrovascular disease	85 (5.3)	7 (4.2)	78 (5.4)	0.712
Heart disease	215 (13.4)	22 (13.3)	193 (13.4)	1.000
Coronary artery disease	142 (8.8)	16 (9.7)	126 (8.7)	0.664
Heart failure	162 (10.1)	15 (9.1)	147 (10.2)	0.785
Valvulopathy	86 (5.3)	6 (3.6)	80 (5.5)	0.364
COPD	88 (5.5)	13 (7.9)	75 (5.2)	0.149
Smoking (present or past)	356 (22.1)	42 (25.5)	314 (21.7)	0.277
Malignancy	94 (5.8)	7 (4.2)	87 (6)	0.482
HIV	33 (2.1)	3(1.8)	30 (2.1)	1.000
Laboratorial findings at ICU admission				
P/F ratio	122 (86;194)	124 (84;191)	122 (87;195)	0.862
White blood cell count (103/µL)	9.9 (7.3;13.7)	9.9 (7.2; 13.7)	10 (7.3;13.7)	0.523
Lactate (mmol/L)	1.5 (1.2; 2)	1.4 (1.1; 1.9)	1.5 (1.2; 2.1)	0.119
Creatinine (mg/dL)	1.0 (0.8;1.6)	0.9 (0.7; 1.6)	1.0 (0.8;1.6)	0.106
CRP (mg/L)	162 (97;242)	152 (83;238)	163 (100;242)	0.129
Outcome				
Death	666 (41.4)	74 (44.8)	592 (41)	0.359
Renal replacement therapy	372 (23.1)	33 (20)	339 (23.5)	0.332
Length of mechanical ventilation	13 (7;24)	11 (7;23)	13 (7; 24)	0.617

Data expressed as median (p25;p75) or n (%).

Supplement Tables S3. Characteristics of patients admitted to the ICU with respiratory failure attributed to COVID-19 or other pulmonary infections (sensitivity analysis including patients without troponin checked)

**Features**	**COVID-19 n = 1609**	**Other pulmonary infections n = 200**	**P value**
**Demographics**			
Age (years)	59 (46;68)	60.5 (42;72)	0.315
Gender			0.013
Male	891 (55.4)	92 (46)	
Female	718 (44.6)	108 (54)	
Body Mass Index (kg/m²)	30.4 (26.4;35.6)	25.9 (21.6;31.2)	<0.001
**Comorbidities**			
Hypertension	910 (56.6)	104 (52)	0.227
Diabetes mellitus	554 (34.4)	62 (31)	0.344
Renal replacement therapy	36 (2.2)	9 (4.5)	0.085
Cerebrovascular disease	85 (5.3)	26 (13)	<0.001
Heart disease	215 (13.4)	51 (25.5)	<0.001
Coronary artery disease	142 (8.8)	31 (15.5)	0.005
Heart failure	162 (10.1)	38 (19)	<0.001
Valvulopathy	86 (5.3)	34 (17)	<0.001
COPD	88 (5.5)	53 (26.5)	<0.001
Smoking (present or past)	356 (22.1)	93 (46.5)	<0.001
Malignancy	94 (5.8)	26 (13)	<0.001
HIV	33(2.1)	17 (8.5)	<0.001
**Laboratory findings at ICU admission**			
D-dimer (µg/mL)	1.5 (0.8; 4.5)	2.4 (1; 5)	0.012
White blood cell count (103/µL)	9.9 (7.3;13.7)	12 (8.8; 15.9)	<0.001
Lactate (mmol/L)	1.5 (1.2; 2)	1.7 (1.2; 3.4)	<0.001
Prothrombin time (seconds)	13.8 (13.1;14.9)	15 (13.8;16.7)	<0.001
Creatinine (mg/dL)	0.9 (0.8; 1.6)	1.2 (0.8;2)	0.045
Fibrinogen (mg/L)	648 (546;748)	541 (375;656)	<0.001
CRP (mg/L)	162 (97;242)	109 (36;213)	<0.001
**Clinical data at ICU admission**			
SOFA score	4 (3;6)	5 (3;8)	0.039
Ventilatory Support			<0.001
Non-invasive or HFNC	402 (25)	21 (10.5)	
Invasive mechanical ventilation	534 (33.2)	83 (41.5)	
Vasopressor	357 (22.2)	75 (37.5)	<0.001
PaO2/FiO2 ratio	122 (86;193)	206 (131;298)	0.000
**Outcomes**			
Renal replacement therapy (new)	372 (23.1)	22 (11)	< 0.001
Pulmonary embolism	334 (20.8)	10 (5)	<0.001
Non survivor	666 (41.4)	54 (27)	<0.001
Composite	930 (57.8)	72 (36)	<0.001
Length of hospital stay	19 (11;31)	13 (9;22)	<0.001
Length of ICU stay	10 (5;20)	4 (1;11)	<0.001
Length of mechanical ventilation	13 (7;24)	6 (4;10.8)	< 0.001
